# Identifying the Demographic and Internet Use Characteristics of Technology-Facilitated Child Sex Offenders Operating in the Australian, U.S. and U.K. General Population

**DOI:** 10.1177/08862605251403620

**Published:** 2026-03-03

**Authors:** Michael Salter, Tyson Whitten, Delanie Woodlock, James Stevenson, Syimah Mat Rani, Deborah Fry

**Affiliations:** 1 University of New South Wales, Sydney, Australia; 2 University of Edinburgh, UK

**Keywords:** offenders/perpetrators, child maltreatment, sexual abuse, child maltreatment, internet and abuse, prevention, child maltreatment, criminology

## Abstract

Research on technology-facilitated child sex offenders outside of forensic and clinical samples is scarce but necessary to inform prevention, early intervention, and investigation. This article describes and compares the demographic characteristics and internet use habits of technology-facilitated child sex offenders sourced from three quota-based samples comparable to the Australian (*n* = 1,945), U.S. (*n* = 1,473), and U.K. (*n* = 1,506) adult male population. The odds (99% CI) of technology-facilitated child sex offending, relative to non-offenders, were calculated for demographic factors (e.g. age, sexual orientation, and number of children in household), hours per day spent online, frequency of engagement in common online behaviours (e.g. sending emails, online messaging, and private video chatting), use of social media platforms (e.g. Facebook, Twitter, and Snapchat), online pornography viewership, and use of privacy software (e.g. The Onion Router [TOR], Telegram, and Element). Logistic regression analysis adjusted for age, educational attainment, household income, and residential location were conducted separately for each sample, with effect sizes formally compared between countries. The study identified significantly higher rates of technology-facilitated offending against children in the United States (10.9%) compared to Australia (7.5%) and United Kingdom (7.0%). Online offenders were between 2 and 3.7 times more likely to work with children and, depending on the jurisdiction, were significantly more likely to be employed, married/living with a partner, have a bachelor’s degree or higher, and live with a child. Across all jurisdictions, technology-facilitated offenders were significantly more sexually active online, including paying for sexual services and content, visiting romance and dating websites, and viewing violent or animal pornography. The article considers the implications of these findings for prevention, early intervention, and more effective detection of technology-facilitated offending, including the role of internet regulation and the financial sector in online child protection.

## Introduction

For the last quarter century, consumer access to the internet has included a significant group of child sex abusers, using various online services and systems to groom and exploit children, produce, distribute, and access child sexual abuse material (CSAM), and create online paedophile communities and networks (Jenkins, 2001). Rates of technology-facilitated child sexual victimisation remain high, posing a significant burden of harm in addition to contact offending (Finkelhor et al., 2024). However, there is limited reliable information about the prevalence and characteristics of perpetration of technology-facilitated child sexual exploitation and abuse, particularly in relation to undetected offenders. To address these knowledge gaps, this article describes and compares the prevalence, demographic characteristics, and internet use habits of technology-facilitated child sex offenders sourced from three samples representative of men in Australia, the United States, and the United Kingdom. The article begins with a literature review situating contemporary research about technology-facilitated child sexual exploitation and abuse within scholarship on the impact of regulatory frameworks on internet behaviour, acknowledging that rates of technology-facilitated sexual offending between countries are likely to be impacted by differences in national legislative and policy frameworks. After an overview of the project methodology, the article describes the prevalence of technology-facilitated child sex offending across the three jurisdictions and the demographic factors and online behaviours associated with online offending across the three samples. The article identifies high levels of technology-facilitated offending against children across the sample and particularly in the United States, as well as demographic and online behavioural characteristics that raise questions about the social determinants of child sexual exploitation and abuse and the potential impact of regulatory frameworks to reduce online sexual harm against children.

## Literature Review

Most studies of technology-facilitated child sex offenders are based on convicted and/or incarcerated offenders, with the primary aim of informing risk assessment, psychological treatment, and recidivism prevention. Researchers have produced a range of categories or typologies of offenders linked to their index offences, incorporating “online” (such as viewing CSAM or engaging in child grooming), “offline” (i.e. contact or in-person), and “mixed” (both online and offline) offending (Lim et al., 2021). However, there is a lack of consensus on the characteristics that distinguish between child sex abusers who do, and do not, use the internet in the commission of their offences. Furthermore, most people who sexually abuse children are never detected by the authorities or held accountable, and hence are not available to study via forensic research. Researchers have used convenience samples to estimate the prevalence of sexual interest and offending against children in the community, which far exceeds the number of people detected for such offences. For example, in an online convenience sample of 8,718 German men, 4.1% indicated a sexual interest in prepubescent children, and 3.2% reported engaging in a sexual offence against a child including 2.4% accessing CSAM (Dombert et al., 2016). In an international online convenience sample of 997 men, 23.1% reported some sexual interest in children and 3.3% reported they had watched CSAM (Ó Ciardha et al., 2022).

The significant minority of men in the community who have viewed CSAM not only signify the level of sexual interest in children in the community but also the widespread online availability of this content. It is important to recognise that the ways in which online services and infrastructure facilitates sexual offending against children is not a natural fact but rather the result of commercial decision-making processes as well as government regulatory frameworks (Keen et al., 2020). Just as contact offending can be enabled by poor child safety practices and situational opportunities to abuse “offline,” technology-facilitated child sexual exploitation and abuse is enabled by internet and electronic service providers who do not implement child safety protocols or engage in proactive child protection measures, who fail to cooperate with law enforcement investigations, and who implement measures such as end-to-end encryption that appeal to offenders seeking to trade CSAM and groom children undetected (Ali et al., 2023; Draper, 2022). Online offenders do not engage in static rates or patterns of offending behaviours but rather they adapt to law enforcement pressure by taking fewer and select opportunities to abuse children in an effort to evade punishment (Balfe et al., 2015; Chopin & Décary-Hétu, 2023). Thus, the ways in which child sex offenders use technology to abuse and exploit children is shaped by a range of factors, including law enforcement pressure, policy and regulatory settings, and the decisions of commercial service providers. It is reasonable to assume that technology-facilitated child sexual exploitation and abuse could be prevented through more effective regulation and other policy initiatives that reduce the utility of technology in the sexual exploitation of children and increase the power of deterrence.

Of the three jurisdictions that are the focus of this article, the United States maintains the most *laissez-faire* approach to internet governance (Carr, 2015). Efforts to regulate the internet in the United States need to contend with robust constitutional free speech protections, as well as the Communications Decency Act of 1996, which provides online service providers with broad civil and criminal immunity for third-party content (Kosseff, 2019). Certain U.S. states have elected to include safety precautions in their jurisdiction, including barring convicted offenders from social networking websites, registration of convicted offenders’ emails and passwords, and prohibiting sexually offensive communication with minors (Chang, 2010).^1^ In contrast, Australia (in 2021) and the United Kingdom (in 2023) have enacted Online Safety Acts. These Acts represent significant developments in the regulation of the internet in both jurisdictions and include provisions relevant to technology-facilitated child sexual exploitation and abuse, including powers for an an online safety body to enforce the regulation of the act in relation to technology companies. A key feature in both Online Safety Acts is a focus on “safety by design” or user protection built into the architecture of online platforms and services (Kira & Shertel Mendes, 2023; Cooray et al., 2023). Both acts also contain stipulations relevant to the removal of CSAM from platforms once a notice had been sent or the material located by mandated proactive searching.

These regulatory measures are focused on the proactive role and responsibility of the technology sector and internet service providers in reducing or disrupting the occurrence of technology-facilitated child sexual exploitation and abuse. The commission of these acts by individuals have been criminalised for some time. For example, the production and distribution of CSAM was criminalised in the three jurisdictions by the early 1980s, prior to the commercialisation of the internet in the 1990s (Salter and Whitten, 2022). Internet-specific offences such as online grooming, luring, and solicitation of children for a sexual purpose were criminalised at a national level in the United Kingdom in 2003, in the United States in 2006, and in Australia in 2009 (Ezioni, 2020). Detection of these offences and enforcement of these provisions frequently hinges on the cooperation (legally mandated or otherwise) of the technology sector. Hence, the presence and relative strength of regulatory measures has direct bearing on the enactment of criminal law and its deterrent effect. Apart from regulation and criminal deterrence, it is worth noting the limited investment in secondary prevention efforts to prevent child sex offending before it occurs, such as programmes targeting people at risk of offending (Kewley et al., 2023).

This article presents an exploratory study that identifies and compares the online behaviours and characteristics of men who engage in technology-facilitated child sex offending in Australia, the United States, and the United Kingdom, controlling for socio-economic factors associated with technology-facilitated child sex offending (age, educational attainment, annual household income, and residential location [Faust et al., 2015]). This article seeks to deepen our understanding of the potential risk factors for technology-facilitated child sex offending in order to inform debates over policy, prevention, and regulatory positions to improve online child protection and safety.

## Methods

This article reports on data drawn from online surveys of men in Australia, the United States, and the United Kingdom regarding their sexual feelings and behaviours towards children. These three countries were selected because they are all high-income, English-speaking countries; therefore, a comparative analysis may provide some insight into the jurisdictional differences that may reduce the prevalence of technology-facilitated child sexual exploitation and abuse. There are high-level similarities in internet use and access across the three jurisdictions, including proportion of adult users, quality and cost of high-speed connectivity, and rates of connectivity to homes and educational institutions (ITU, 2023). At least 95% of the adult population in these three countries are internet users, with a distinctive skew towards younger users (ITU, 2023).

### Data

Data were drawn from an online survey examining the prevalence and factors associated with men’s sexual attitudes, feelings, and behaviours towards children. The survey was administered from November to December 2022 by CloudResearch (https://www.cloudresearch.com), an online recruitment and survey company with access to an international pool of over 1.5 million participants. Quota sampling was performed until we obtained three samples of around 1,500 adult men comparable to the Australian, U.K., and U.S. male populations in terms of age, residential region, annual household income, and educational attainment. Participants were excluded if they indicated that they were born female, did not identify as male, answered questions dishonestly, or failed the attention check. Data were weighted using iterative proportional fitting based on six demographic factors (race, marital status, employment status, age, annual household income, and educational attainment) sourced from each country’s respective 2021 census. Ethics approval was provided by the Human Research Ethics Committee at the University of New South Wales (HC220317).

### Measures

#### Technology-Facilitated Child Sex Offending

Participants were asked if they had engaged in any of the following behaviours while over the age of 18 years: (a) knowingly and deliberately viewed pornographic material containing people below the age of 18; (b) flirted or had sexual conversations with a person below the age of 18 online; (c) engaged in a sexually explicit webcam interaction with a person below the age of 18; and (d) paid for online sexual interactions, images, or videos involving a person below the age of 18. Respondents who indicated yes to any of these questions were coded as having engaged in technology-facilitated child sex offending.

#### Demographic Characteristics

Participants reported their sexual orientation (0 = *not heterosexual*; 1 = *heterosexual*), if they had ever had sex with men (0 = *no*; 1 = *yes*), current relationship status (0 = *single, widowed, divorced, or separated*; 1 = *married or living with partner*), employment over the last 3 months (0 = *unemployed*; 1 = *casual, part-time, or full-time employment*), highest educational attainment (0 = *did not obtain a bachelor’s degree*; 1 = *bachelor’s degree or higher*), number of children living in the household (0 = *none*; 1 = *one or more*), if their current work involves contact with children (0 = *does not work with children*; 1 = *works with children*), age (1 = 18–34 years; 2 = 35–64 years; 3 = 65 years or older), total U.S. standardised household income before taxes during the last 12 months (1 = *less than US$25,000 equivalent*; 2 = *between US$25,000–US$99,999*; 3 = *US$100,000 or more*), and residential location (1 = *city*; 2 = *suburb*; 3 = *rural or regional*).

#### Online Pornography Habits

Respondents indicated (0 = *no*; 1 = *yes*) if, while over the age of 18 years, they: (a) knowingly and deliberately viewed pornography involving sex between humans and animals; (b) watched pornography that included sex with violence or force; (c) purchased online sexual services from another person; (d) were ever approached online by an adult offering sexual images, videos, or content; and (e) were ever approached online by a person under 18 years offering sexual images, videos, or content.

#### Frequency of Online Activities

Participants reported how often (1 = *never*, 2 = *less than monthly*, 3 = *monthly*, 4 = *weekly*, 5 = *daily*) they engaged in 13 common internet activities: (a) using search engines to browse or look for information; (b) sending emails; (c) using social media; (d) engage in online blogs; (e) shopping online; (f) online banking; (g) online messaging; (h) private video chatting; (i) livestreaming self; (j) streaming movies or shows to personal devices; (k) using romance websites or dating apps; (l) online gaming; and (m) watching online pornography.

#### Social Media Platform Use

Participants indicated (0 = *no*, 1 = *yes*) if they currently used any of the following social media platforms: (a) YouTube; (b) Instagram; (c) Facebook; (d) Snapchat; (e) Facebook messenger; (f) TikTok; (g) WhatsApp; (h) Twitter; (i) Discord; (j) Skype; and (k) Viber.

#### Privacy Software and Anonymity Tools

Respondents reported (0 = *no*, 1 = *yes*) if they used any of the following services to prevent tracking and surveillance of their online activities: (a) TOR; (b) Virtual Private Network (VPN); (c) Telegram; (d) Signal; (e) WhatsApp; (f) Element; (g) Hive; (h); and (i) Private relay (Safari). Participants also reported if they (a) currently own any cryptocurrencies (e.g. Bitcoin, Ethereum, and XRP), and if they had (b) ever used cryptocurrencies to purchase items or services online.

### Analytical Strategy

Weighted descriptive statistics with 95% confidence intervals (95% CIs) regarding the prevalence of technology-facilitated child sex offending, demographic characteristics, online pornography habits, frequency of online activities, use of social media platforms, and use of privacy software and anonymity tools were presented separately for the Australian, U.K., and U.S. samples. Next, univariate weighted logistic regression analysis was conducted to describe the unadjusted association between technology-facilitated child sex offending and the included variables. A series of multivariable weighted logistic regression analyses were also calculated to identify the association between technology-facilitated child sex offending and each variable independent of age, educational attainment, annual household income, and residential location. Regression analyses were conducted separately for each country. To account for multiple comparisons, associations were considered significant if *p*-values were *p* < .01.

Results were calculated using survey weights, with robust standard errors (Lumley 2004). All assumptions underpinning logistic regression analysis were met (Tabachnick & Fidell, 2013). Odds ratios (ORs) and 99% CIs were reported as measures of effect size and precision of the association between the covariates and outcome variables. Between-country differences in effect sizes were conducted using the methodology outlined by Altman (2003) and reported if differences were significant at *p* < .05 (two tailed). Analyses were conducted using IBM SPSS 29 (IBM Corp, 2022).

## Results

Table 1 presents the descriptive statistics for the Australian (*n* = 1,939), U.K. (*n* = 1,506), and U.S. (*n* = 1,473) samples. There were substantial differences between the samples regarding demographic characteristics, online pornography viewership, internet use frequency, social media platform use, and privacy software use. Importantly, significantly more men from the United States (10.9%) than Australia (7.5%) and United Kingdom (7.0%) had engaged in some form of technology-facilitated child sexual exploitation (*x*^2^ = 12.26, *p* *<* .01). The magnitude of this difference was greatest for those who had paid for online sexual interactions, images, or videos involving a child (*x*^2^ = 24.38, *p* *<* .001), followed by those who had engaged in a sexually explicit webcam interaction with a child (*x*^2^ = 21.82, *p* *<* .001), deliberately watched pornographic material containing children (*x*^2^ = 14.00, *p* < .001), and had sexual conversations with children online (*x*^2^ = 6.74, *p* < .05). By contrast, similar proportions of Australian and U.K. men deliberately watched pornography involving children (*x*^2^ = 0.40, *p* = .53), had sexual conversations with children online (*x*^2^ = 0.60, *p* = .44), had sexually explicit webcam interactions with children (*x*^2^ = 0.66, *p* = .42), and paid for online sexual content of children (*x*^2^ = 0.31, *p* = .58).

**Table 1.** Weighted Descriptive Statistics (95% CI).

Measures	Australia (*n* = 1,938)	United Kingdom (*n* = 1,505)	United States (*n* = 1,473)
Tech-Facilitated Child Sexual Exploitation and Abuse (TF-CSEA)			
Any TF-CSEA	7.5% (6.3%, 9.0%)	7.0% (5.7%, 8.7%)	10.9% (9.3%, 12.9%)
Sexual conversations	4.3% (3.4%, 5.5%)	3.7% (2.7%, 5.0%)	5.9% (4.7%, 7.4%)
Watch child sexual abuse material	2.5% (1.9%, 3.4%)	2.9% (2.1%, 4.2%)	5.1% (4.0%, 6.6%)
Sexually explicit webcam	1.8% (1.2%, 2.6%)	1.4% (0.9%, 2.2%)	4.2% (3.2%, 5.5%)
Paid for sexual content	1.7% (1.2%, 2.5%)	2.0% (1.3%, 3.0%)	4.8% (3.7%, 6.2%)
Demographic Characteristics			
Heterosexual	92.7% (91.1%, 93.9%)	92.7% (91.0%, 94.0%)	93.5% (92.0%, 94.7%)
Had sex with men	15.3% (13.4%, 17.4%)	16.6% (14.6%, 18.9%)	17.5% (15.4%, 19.8%)
Married or living with partner	58.3% (55.5%, 61.0%)	62.7% (59.8%, 65.6%)	59.5% (56.6%, 62.3%)
Employed	68.5% (65.8%, 71.1%)	63.5% (60.6%, 66.4%)	64.0% (61.2%, 66.8%)
Child in household	33.9% (31.4%, 36.4%)	29.6% (27.0%, 32.3%)	34.0% (31.3%, 36.8%)
Works with children	16.3% (14.6%, 18.1%)	15.4% (13.4%, 17.6%)	16.6% (14.5%, 18.9%)
Bachelor’s degree or higher	38.9% (36.2%, 41.6%)	41.5% (38.6%, 44.5%)	31.2% (28.7%, 33.8%)
Residential location			
City	31.2% (28.7%, 33.7%)	32.6% (29.8%, 35.4%)	38.5% (35.8%, 41.4%)
Suburbs	51.8% (49.0%, 54.5%)	37.6% (34.8%, 40.5%)	39.0% (36.2%, 41.9%)
Regional or rural	17.1% (15.2%, 19.2%)	29.8% (27.2%, 32.5%)	22.5% (20.2%, 24.9%)
Age			
18–34 years	32.9% (30.5%, 35.5%)	28.1% (25.5%, 30.9%)	30.1% (27.4%, 32.9%)
35–64 years	46.9% (44.2%, 49.7%)	49.7% (46.8%, 52.6%)	50.0% (47.1%, 52.9%)
65 years and older	20.1% (18.1%, 22.4%)	22.2% (19.8%, 24.8%)	19.9% (17.8%, 22.2%)
Household income			
Less than US$25,000	28.9% (26.2%, 31.7%)	19.0% (16.8%, 21.3%)	17.4% (15.2%, 19.8%)
US$25,000–US$99,999	46.7% (44.0%, 49.4%)	69.5% (66.9%, 71.9%)	48.8% (45.9%, 51.7%)
US$100,000 or more	24.4% (22.2%, 26.9%)	11.5% (10.3%, 12.9%)	33.8% (31.1%, 36.7%)
Online Pornography Habits			
Watches violent or rough porn	13.0% (11.3%, 15.0%)	9.4% (7.7%, 11.4%)	10.4% (8.8%, 12.3%)
Watches bestiality	4.2% (3.2%, 5.5%)	4.1% (3.1%, 5.3%)	7.4% (6.1%, 9.1%)
Purchase sexual content	6.6% (5.5%, 7.9%)	10.9% (9.2%, 12.8%)	15.5% (13.5%, 17.8%)
Approached by adult	20.8% (18.7%, 23.1%)	17.0% (15.0%, 19.3%)	25.0% (22.6%, 27.6%)
Approached by child	6.7% (5.4%, 8.2%)	5.4% (4.2%, 6.9%)	10.1% (8.4%, 12.0%)
Mean Frequency of Online Activities			
Browse online	4.69 (4.65, 4.73)	4.70 (4.65, 4.74)	4.61 (4.57, 4.66)
Send emails	4.32 (5.26, 4.37)	4.31 (4.25, 4.37)	4.10 (4.02, 4.17)
Social media	4.26 (4.18, 4.33)	4.16 (4.07, 4.24)	4.17 (4.08, 4.25)
Online blogs	2.96 (2.87, 3.05)	2.83 (2.74, 2.93)	2.94 (2.85, 3.04)
Online shopping	2.90 (2.84, 2.96)	3.08 (3.01, 3.14)	3.00 (2.92, 3.07)
Online banking	4.03 (3.98, 4.09)	4.06 (4.00, 4.12)	3.69 (3.61, 3.77)
Online messaging	3.82 (3.74, 3.91)	3.83 (3.75, 3.92)	3.68 (3.59, 3.77)
Private video chatting	2.85 (2.77, 2.94)	2.79 (2.70, 2.88)	2.80 (2.71, 2.89)
Livestream self	2.23 (2.15, 2.32)	2.19 (2.09, 2.28)	2.57 (2.48, 2.67)
Streaming videos	3.76 (3.67, 3.84)	3.57 (3.48, 3.66)	3.69 (3.60, 3.78)
Romance/dating websites	1.89 (1.82, 1.96)	1.90 (1.81, 1.99)	2.18 (2.09, 2.27)
Online gaming	2.53 (2.45, 2.62)	2.38 (2.29, 2.47)	2.66 (2.56, 2.76)
Online porn	2.60 (2.51, 2.68)	2.41 (2.32, 2.50)	2.61 (2.51, 2.70)
Use of Social Media Platforms			
YouTube	77.2% (74.9%, 79.4%)	71.6% (68.9%, 74.2%)	72.2% (69.5%, 74.8%)
Instagram	44.0% (41.4%, 46.7%)	41.6% (38.8%, 44.5%)	46.2% (43.3%, 49.1%)
Facebook	71.0% (68.5%, 73.5%)	64.6% (61.7%, 67.3%)	71.1% (68.4%, 73.7%)
Snapchat	26.7% (24.4%, 29.1%)	23.4% (20.9%, 26.0%)	30.4% (27.8%, 33.2%)
Facebook messenger	49.4% (46.7%, 51.2%)	42.1% (39.2%, 45.0%)	46.2% (43.3%, 49.1%)
Tik Tok	31.3% (28.9%, 33.9%)	26.8% (24.3%, 29.5%)	34.5% (31.8%, 37.4%)
WhatsApp	38.2% (35.6%, 40.9%)	61.0% (58.1%, 63.8%)	24.3% (21.9%, 26.9%)
Twitter	30.9% (28.5%, 33.5%)	35.1% (32.4%, 37.9%)	33.1% (30.4%, 35.8%)
Discord	16.1% (14.2%, 18.1%)	10.1% (8.5%, 12.0%)	11.1% (9.4%, 13.1%)
Skype	13.0% (11.3%, 14.9%)	11.1% (9.3%, 13.1%)	12.2% (10.5%, 14.2%)
Viber	5.2% (4.0%, 6.7%)	2.8% (2.0%, 3.9%)	2.9% (2.1%, 4.1%)
Use of Privacy Software and Anonymity Tools			
Any privacy software	48.7% (46.0%, 51.4%)	54.9% (51.9%, 57.8%)	48.3% (45.4%, 51.2%)
TOR	4.7% (3.7%, 5.9%)	3.2% (2.4%, 4.3%)	8.3% (6.8%, 10.0%)
VPN	25.0% (22.7%, 27.4%)	21.7% (19.4%, 24.2%)	26.0% (23.5%, 28.6%)
Telegram	10.8% (9.3%, 12.4%)	10.5% (8.9%, 12.5%)	16.1% (14.1%, 18.4%)
Signal	7.9% (6.6%, 9.5%)	4.7% (3.6%, 6.0%)	6.4% (5.2%, 7.9%)
WhatsApp	25.7% (23.4%, 28.1%)	37.2% (34.4%, 40.1%)	24.3% (21.8%, 26.9%)
Element	2.1% (1.5%, 2.9%)	1.8% (1.2%, 2.7%)	3.7% 92.8%, 4.9%)
Hive	4.0% (3.1%, 5.3%)	2.7% (1.9%, 3.8%)	3.4% (2.5%, 4.6%)
Private relay (Safari)	3.0% (2.2%, 4.0%)	3.1% (2.2%, 4.4%)	4.4% (3.4%, 5.6%)
Owns cryptocurrency	23.9% (21.8%, 26.2%)	19.8% (17.6%, 22.2%)	33.3% (30.6%, 36.1%)
Uses cryptocurrency	9.3% (8.0%, 10.8%)	9.6% (8.0%, 11.5%)	21.6% (19.3%, 24.1%)

*Note.* TOR = The Onion Router; VPN = Virtual Private Network.

**Figure 1.**
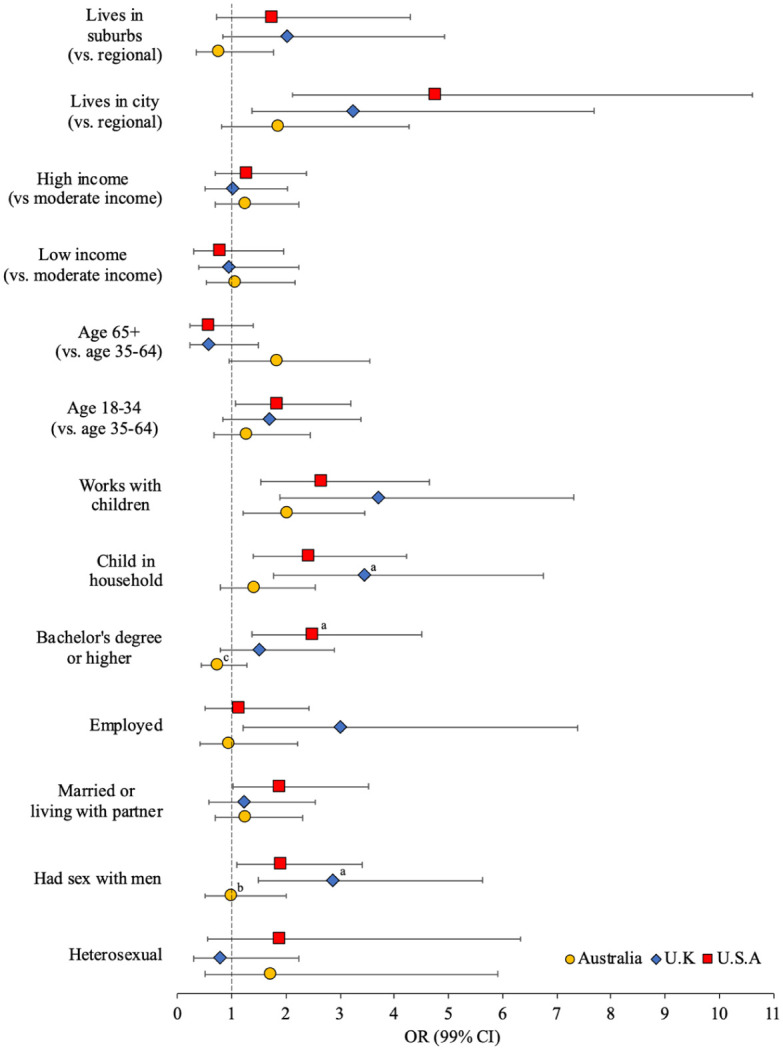
Adjusted odds (99% CI) of tech-facilitated child sexual exploitation by demographic characteristics (superscript indicates significant difference in effect size between Australia^a^, U.K.^b^, and/or U.S.^c^ sample).

Figure 1 presents the multivariable associations between technology-facilitated child sex offending and demographic characteristics (weighted unadjusted and adjusted ORs [99% CI] presented in Supplemental Table S1). Across the three samples, men who engaged in technology-facilitated child sex offending, relative to those who did not, were significantly more likely to work with children (OR = 2.03–3.71). Technology-facilitated child sex offending was significantly associated with employment (OR = 3.00) in the U.K. sample only, whereas being married or living with a partner (OR = 1.90), having a bachelor’s degree or higher (OR = 2.49), and being aged between 18 and 34 years (OR = 1.84) were only significant in the U.S. sample. In both the United Kingdom and United States, men who engaged in technology-facilitated child sex offending were significantly more likely to have had sex with men (OR = 2.88–1.92), have a child in the household (OR = 3.45–2.43), and live in the city (OR = 3.25–4.76). Effect size comparisons indicate that the odds of technology-facilitated child sex offending were significantly greater for U.K. than Australian men who had ever had sex with men (*d* = 1.06 [*se* = 0.49], *p* < .05) or had a child in the household (*d* = 0.89 [*se* = 0.46], *p* < .05), whereas the odds were significantly greater for U.S. than Australian men who had a bachelor’s degree or higher (*d* = 1.20 [*se* = 0.40], *p* < .01).

**Figure 2.**
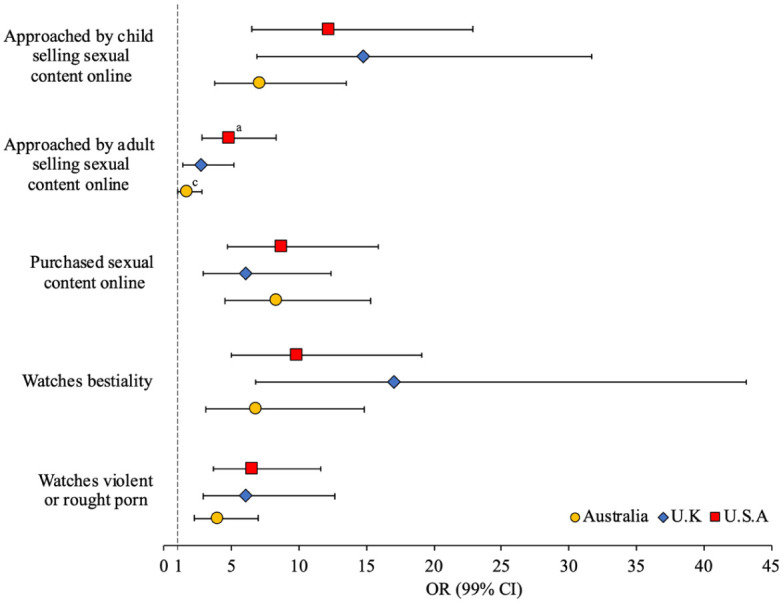
Adjusted odds (99% CI) of tech-facilitated child sexual exploitation by online pornography habits (superscript indicates significant difference in effect size between Australia^a^, U.K.^b^, and/or U.S.^c^ sample).

Figure 2 presents the adjusted odds of technology-facilitated child sex offending by online pornography habits (weighted unadjusted and adjusted ORs [99% CI] presented in Supplemental Table S2). Regardless of country, men who engage in technology-facilitated child sex offending were 7.08 to 14.75 times more likely to report that they had been approached by a child selling sexual content online, 6.73 to 17.05 times more likely to watch bestiality, 5.98 to 8.67 times more likely to purchase sexual content and services online, and 3.90 to 6.50 times more likely to watch violent and rough pornography. Having been approached by an adult selling sexual content online was associated with technology-facilitated child sex offending in the U.K. and U.S. samples only. The odds of child sex offending were significantly greater for U.S. than Australian men who had been approached by an adult selling sexual content online (*d* = 1.07 [*se* = 0.38], *p* < .01).

**Figure 3.**
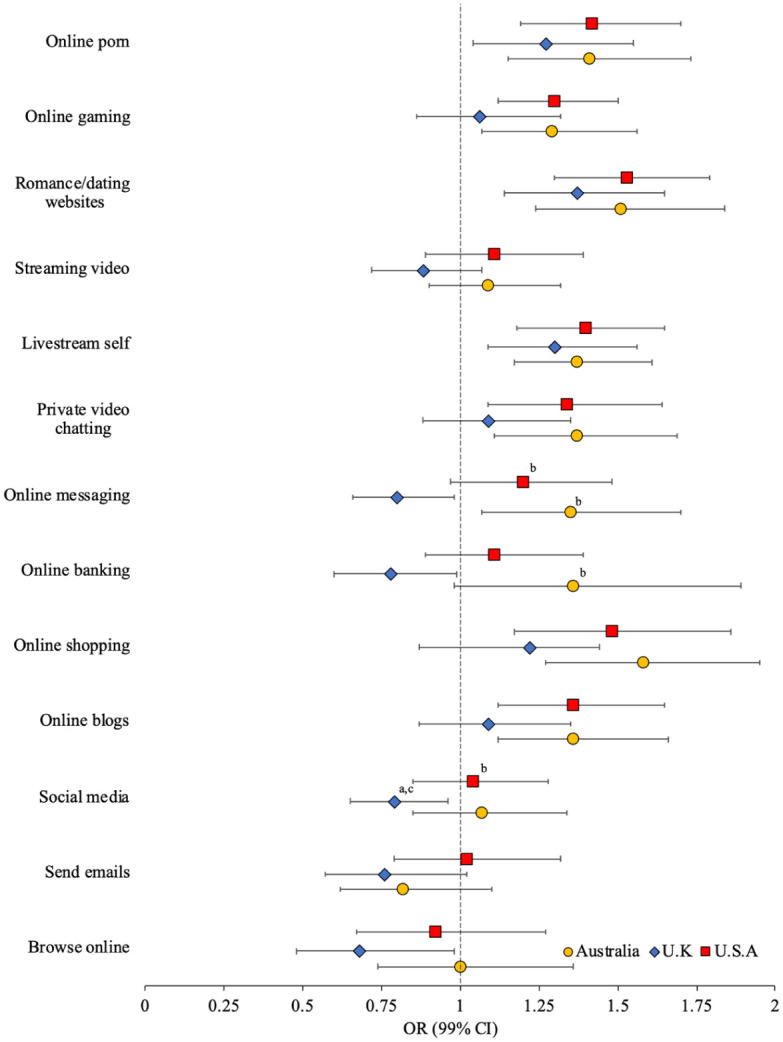
Adjusted odds (99% CI) of tech-facilitated child sexual exploitation by frequency of online activities (superscript indicates significant difference in effect size between Australia^a^, U.K.^b^, and/or U.S.^c^ sample).

The adjusted odds of technology-facilitated child sex offending by frequency of online activities are presented in Figure 3 (weighted unadjusted and adjusted ORs [99% CI] presented in Supplemental Table S3). Across all samples, technology-facilitated child sex offending was significantly associated with more frequent engagement with online pornography (OR = 1.27–1.42), romance and dating websites (OR = 1.37–1.53), and livestreaming oneself (OR = 1.30–1.40). In the Australian and U.S. samples, technology-facilitated child sex offending was significantly associated with more frequent online gaming (OR = 1.29–1.30), private video chatting (OR = 1.34–1.37), online shopping (OR = 1.48–1.58), and online blogging (OR = 1.36–1.36). By contrast, in the U.K. sample, technology-facilitated child sex offending was significantly associated with less frequent engagement in online browsing (OR = 0.68), social media (OR = 0.79), online banking (OR = 0.78), and online messaging (OR = 0.80). Effect size comparisons indicate that less frequent social media use (*d* = 0.30 [*se* = 0.15], *p* < .05; *d* = 0.27 [*se* = 0.14], *p* < .05), online banking (*d* = 0.56 [*se* = 0.21], *p* < .01; *d* = 0.35 [*se* = 0.17], *p* < .05), and online messaging (*d* = 0.52 [*se* = 0.16], *p* < .001; *d* = 0.41 [*se* = 0.15], *p* < .01) were associated with significantly higher odds of technology-facilitated child sex offending for U.K. men than for Australian and U.S. men, respectively.

**Figure 4.**
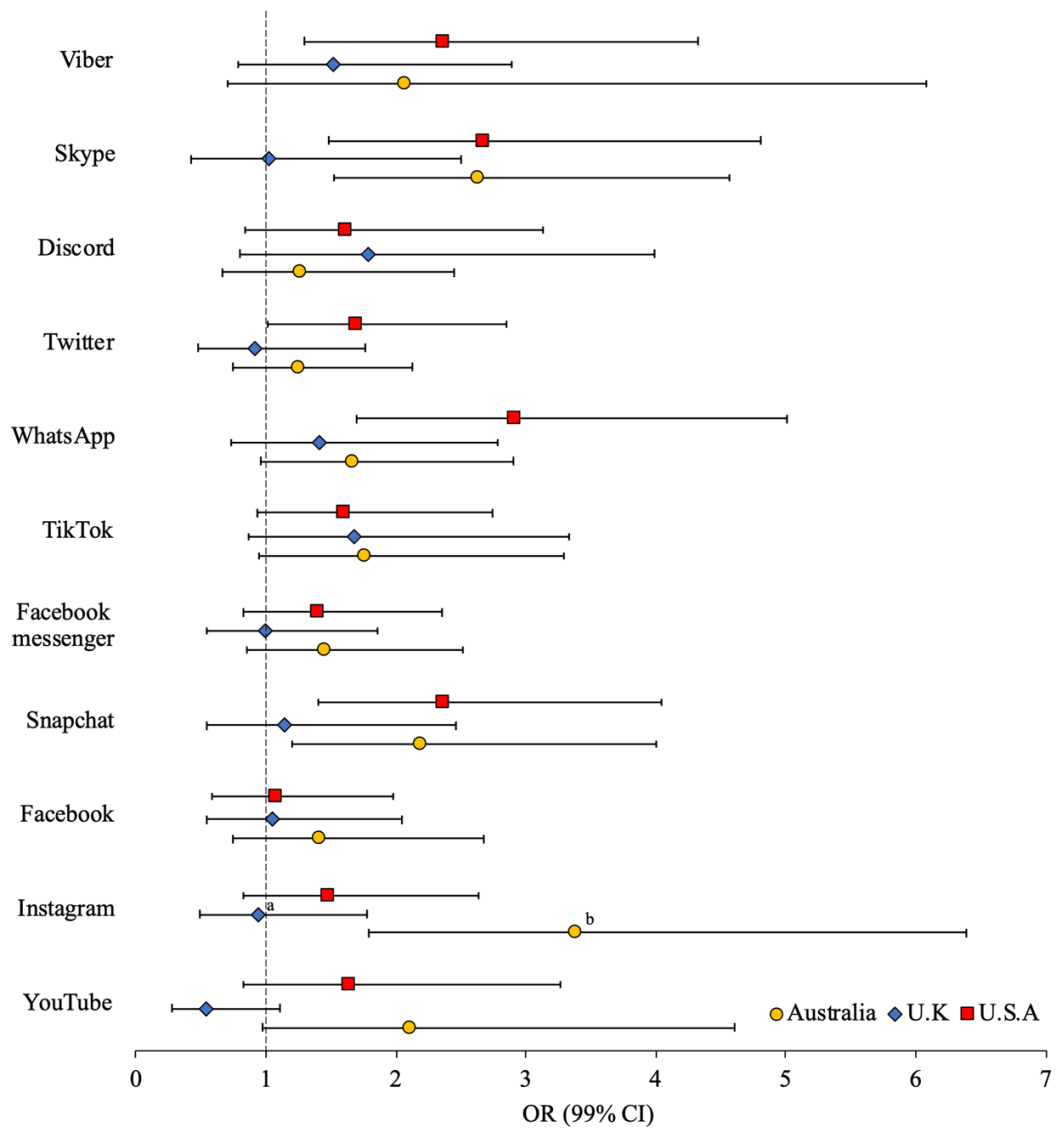
Adjusted odds (99% CI) of tech-facilitated child sexual exploitation by use of social media platforms (superscript indicates significant difference in effect size between Australia^a^, U.K.^b^, and/or U.S.^c^ sample).

Figure 4 presents the adjusted associations between technology-facilitated child sex offending and use of social media platforms (weighted unadjusted and adjusted ORs [99% CI] presented in Supplemental Table S4). Notably, no significant associations were observed for the U.K. sample. Men from Australia and the United States who engaged in technology-facilitated child sex offending were 2.19 to 2.37 times more likely to use Snapchat and 2.63 to 2.67 times more likely to use Skype. Instagram use was significantly associated with technology-facilitated child sex offending (OR = 3.38) among Australian men only; these odds were significantly greater than for men from the United Kingdom (*d* = 0.1.28 [*se* = 0.46], *p* < .01). WhatsApp (OR = 2.92), Twitter (OR = 1.70), and Viber (OR = 2.37) were only significant among men from the United States.

**Figure 5.**
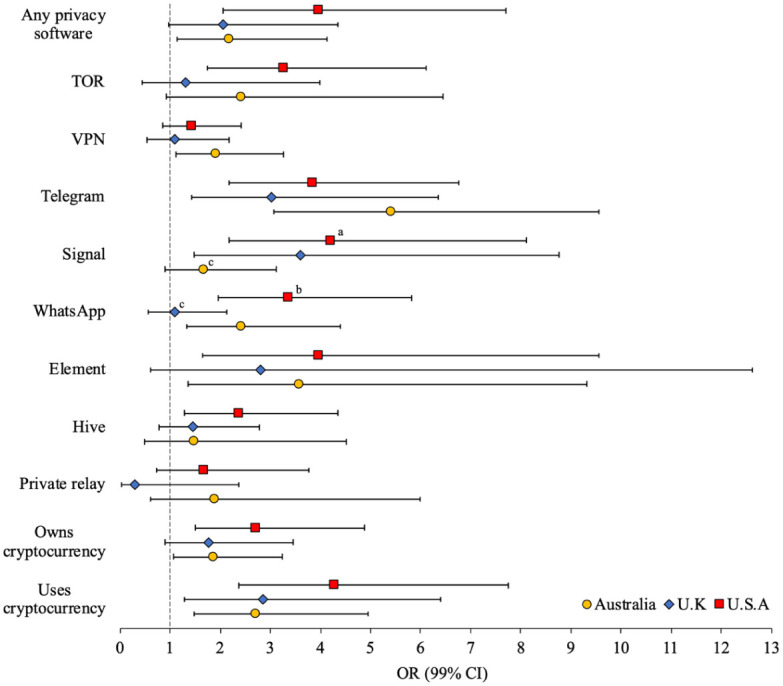
Adjusted odds (99% CI) of tech-facilitated child sexual exploitation by use of privacy software and anonymity tools (superscript indicates significant difference in effect size between Australia^a^, U.K.^b^, and/or U.S.^c^ sample).

The adjusted odds of technology-facilitated child sexual exploitation and use of privacy software and anonymity tools are presented in Figure 5 (weighted unadjusted and adjusted ORs [99% CI] presented in Supplemental Table S5). Men from Australia, the United Kingdom, and the United States who engaged in technology-facilitated child sex offending were 3.03 to 5.40 times more likely to use Telegram and 2.71 to 4.28 times more likely to use cryptocurrencies for online purchases. Technology-facilitated child sex offenders from Australia and the United States were 2.18 to 3.97 times more likely to use any privacy software, 2.42 to 3.37 times more likely to use WhatsApp, 3.57 to 3.96 times more likely to use Element, and 1.86 to 2.72 times more likely to own cryptocurrency. Signal was significantly more likely to be used by offenders from the United Kingdom (OR = 3.61) and United States (OR = 4.21). Technology-facilitated child sex offenders from Australia were 1.92 times more likely to use a VPN, whereas offenders from the United States were 3.27 times more likely to use TOR and 2.36 times more likely to use Hive. The odds that men who engage in technology-facilitated child sex offending use Signal was significantly greater for those from the United States than Australia (*d* = 0.92 [*se* = 0.46], *p* < .05), while the odds that these men would use WhatsApp was greater for those from the United States than the United Kingdom (*d* = 1.12 [*se* = 0.44], *p* < .01).

## Discussion

This is the first comparative study to examine the demographic characteristics and internet use among a representative sample of men who report technology-facilitated sexual offending behaviours against children in three countries. This study found that a substantial minority of men in the community have engaged in some form of technology-facilitated child sexual exploitation and abuse, with significant differences in perpetration prevalence between the United States (10.9%) compared to Australia (7.5%) and the United Kingdom (7.0%). Significantly more men in the United States had engaged in some form of commercial online sexual exploitation (such as paying for sexual interactions, images, or videos involving a child), sexually explicit webcam with a child (a form of abuse that typically has a commercial element, see Napier et al., 2020), viewing CSAM or having sexual conversations with a child. Similar proportions of men in Australia and the United Kingdom had viewed CSAM, had sexual conversations with a child, engaged in sexually explicit conversations with a child, or paid for online sexual content of children.

The article has identified a number of demographic and behavioural characteristics that differentiate men who offended against children online from men who do not. Across all three jurisdictions, men who engage in technology-facilitated child sex offending, compared to men who did not, were more likely to work with children. The strength of other characteristics associated with online offending varied between countries. In the United Kingdom, online offenders were more likely to be employed, live in the city, have a child in the household, and have had sex with men. In the United States, they were more likely to be young, married/partnered, educated at a bachelor level or above, and also have a child in the household, live in the city, and have had sex with men. It is important to note that sexual orientation was not associated with offending, and it is not uncommon for heterosexually identifying men to have had sex with men in the past (Twenge et al., 2016). In the current study, the proportion of heterosexual men who have had sex with men was 10.2% (95% CI [8.5%, 12.2%]) in Australia, 11.3% ([9.5%, 13.4%]) in the United Kingdom, and 13.1% ([11.1%, 15.3%]) in the United States.

The findings paint a picture of an offender who appears outwardly respectable and trusted, with access to children. A study of extrafamilial contact offenders found that such offenders are particularly difficult to detect and may offend against children for a prolonged period of time, compared to offenders who are more demonstrably antisocial (Nicol et al., 2022). Accordingly, so-called protective factors against criminality and recidivism such as “employment, stable relationships, and an apparent ability to self-regulate” may in fact “facilitate detection evasion rather than act as a barrier to abuse” (Nicol et al., 2022, p. 14). This finding is also relevant in the context of ongoing debates over the role of “good character” evidence in sentencing in child sexual assault matters. While there is evidence that survivors of child sexual abuse and the general public believe that “good character” indicates “greater culpability as they [child abusers] were hiding behind a ‘cloak of respectability’ but knew the offence was wrong” (Nicholls et al., 2012, p. 56), judges tend to give far more weight to “good character” evidence than jurors (Warner et al., 2018). In Australia, some legal associations have rejected proposals to prohibit courts from considering a child sex offender’s “good character” in sentencing (Gore, 2024). The findings of this study present further evidence that a positive public image and reputation are common characteristics of technology-facilitated child sex offenders.

Technology-facilitated child sex offenders had a heightened engagement with online sexual services and content. Across the three samples, online offenders reported significantly more frequent engagement with online pornography than non-offenders, and were more likely to view bestiality material as well as violent and rough pornography. They were significantly more likely to pay to view sexual content and material online. Other studies have also documented a significant level of engagement with adult pornography, including paying for content, amongst CSAM offenders (Henshaw et al., 2017). However, as Quayle notes, “[t]he fact that many of those who are identified or self-identify as using Child Sexual Exploitation Material do not engage exclusively with this content is of interest but largely unexplored.” In one study of 268 men convicted of CSAM offences in Canada, 90% were also found to possess adult pornography, and 87% (of the sub-sample of 240 men where the authors could assess their pornography collection) had pornography with paraphilic themes, such as sadism, masochism, or bestiality (Seto & Eke, 2017).

Our study finds that engagement with adult content and sexual interest in paraphilic themes are higher amongst technology-facilitated child sex offenders than non-offending men in the general community. The online offending scholarship to date has drawn mixed conclusions about the significance of this overlap between online offending against children (particularly CSAM), and paraphilic interest and risk of contact offending (Henshaw et al., 2017). However, previous studies have identified a link between heightened sexual interest or “hypersexuality,” sexual interest in children and other paraphilic interests such as bestiality (Wurtele et al., 2018) while highly traded CSAM series includes recordings of child torture and children forced into sexual contact with animals (Seto et al., 2018; Salter et al., 2025). Our study provides further evidence that heightened sexual interest or preoccupation (evident in this study through greater engagement with adult pornography) may be a contributor to technology-facilitated sexual offending against children.

This article provides additional evidence that technology-facilitated child sex offenders are engaged in other online services relating to sex and sexuality, such as the use of dating and romance sites. While this correlation may appear relatively innocuous, it is important to recognise that dating and romance applications are increasingly recognised as sites of risk for child sexual exploitation and abuse. Elsewhere, we have analysed the increased odds and frequency of dating and romance site use amongst child sex offenders in our data, showing that child sex offenders are disproportionately active on these sites (Salter et al., 2025). Our findings correlate with other studies, showing that social media users are complaining about the presence of child sex abusers on romance and dating sites (Levine et al., 2022). A recent large-scale Australian survey found that a considerable proportion of adults who use online dating and romance sites have been approached by a stranger asking for images of their children, including nude and sexual images (Teunissen et al., 2022). A review of CSAM production prosecutions in Australia identified a small group of single mothers who sexually exploited their children at the behest of online “boyfriends” (Author, 2021). This study contributes to the cumulative evidence of the role of dating and romance apps in child sexual exploitation and the need for proactive and preventative measures to reduce the risk posed by an over-representation of online offenders on these sites.

Across the three samples, men who commit online offences against children were significantly more likely to report that they had been approached by a child selling sexual content online, while being approached by an adult selling sexual content online was associated with offenders in the U.K. and U.S. samples. These findings require further exploration and research, particularly in relation to the potential behaviour of minors (if, indeed, they are minors or adult offenders posing as minors) in self-producing and selling images, videos, or other online interactions for profit, as well as the online contexts in which online offenders are interacting with minors or adults selling sexual services. A nationally representative online survey of young Americans aged 18 to 28 found that 1.7% reported ever receiving money for the provision of online sexual interactions or content while a minor (Finkelhor et al., 2022). A recent study by the Stanford Internet Observatory found large networks of social media accounts, ostensibly operated by children, openly advertising self-generated sexual and nude images and videos for sale, with Instagram the most significant platform facilitating the exchange of money for CSAM (Thiel et al., 2023). Other platforms named in the study included Snapchat, Twitter, Discord, and Telegram, pointing to a mixture of child-focused and encrypted platforms facilitating the production and exchange of CSAM between minors and adult offenders. In our study, the social media sites associated with online offending varied between jurisdictions, and it is important to recognise that men who engage in technology-facilitated abuse may be particularly active online, and using online services and platforms in ways unrelated to their offending. Nonetheless, technology-facilitated offenders in our study were disproportionately active on sites with a majority youth audience, such as Snapchat (in Australia and the United States) and Instagram (in Australia). Further research is necessary into the circumstances in which child sex offenders are intersecting with children who may be seeking to sell sexual material or interactions online.

Given their levels of online offending, perhaps it is unsurprising that, across the three samples, technology-facilitated child sex offenders were three to five times more likely to use the encrypted social media app Telegram, and far more likely to use cryptocurrency to pay for online purchases. Offender preferences for other encrypted and privacy services varied between jurisdictions, including the encrypted social media apps WhatsApp and Element (in Australia and the United States), Signal (in the United States and United Kingdom), VPNs (in Australia), and the TOR browser (or “dark web”) and the encrypted social media app Hive (in the United States). These findings provide clear evidence of an offender preference for encrypted social media apps and other privacy services, and further support for the characterisation of online offenders as premeditated and deliberative in their illegal behaviour. These findings are also relevant to ongoing debates in the United States, Australia, and elsewhere regarding the implementation of encryption on social media sites, and the obligations of encrypted apps and services to ensure that their services are not being used to facilitate the sexual abuse of children.

The current study is the first multi-country study that explores the prevalence, demographics, and internet use characteristics of men who report technology-facilitated child sexual exploitation offending in Australia, the United Kingdom, and the United States. This design enhances both the exploration and generalisability of the findings across contexts, which is critical for developing both universal and targeted prevention strategies.

Despite this, there are several limitations that should also be considered. This is an international comparative study of online offending and the article used 18 as the age of consent for all offences. While 18 is often the age of consent in technology-facilitated child sexual abuse criminal law, there is variability across jurisdictions for some offences, and there has been variation over time, such that behaviour that is criminal today may not have been an offence 20 years ago. It is not possible or feasible to sensitise the survey instrument to these legal nuances. As a result, it is possible that some lawful activity has been categorised in this survey as unlawful. Nonetheless, we have conducted a secondary analyses of our data to examine this prospect, which did not support the proposition that our offending findings have been unduly inflated (Author, 2025).

Where a survey respondent reported criminal behaviour, we did not ask further information about an offending incident (e.g. the specific age or sex of the child victim) due to a range of concerns. These concerns included the possibility that gathering data on offending incidents might increase the perception of risk and therefore attrition from the survey amongst offending participant, and such information could reach the mandatory reporting threshold in our jurisdiction, obliging us to breach the confidentiality provisions of the research project. As a result, the survey did not gather specific information about incidents of offending that might inform a more nuanced analysis of risk or offending patterns. This survey is based on self-report measures, and self-reported data of illegal behaviours can introduce desirability or recall bias, which is likely to underestimate the true prevalence of online offending. We sought to minimise this risk by asking behaviourally specific questions without judgement. The cross-sectional design of the study also limits the ability to establish the temporal ordering between internet use and sexual feelings and behaviours towards children; longitudinal studies would be more definitive in determining pathways of offending and internet use and other online behaviours. Finally, this study focuses specifically on men since they are by far the main perpetrators of both online and offline child sexual exploitation and abuse. Future research should explore whether these same patterns of offending exist for female perpetrators.

### Conclusion

One of the striking findings of this study is the significantly higher rate of online offending against children in the United States compared to Australia and the United Kingdom. This article highlighted how these three countries have very different legislative and policy landscapes in relation to online regulation and suggests this may be a contributing factor to the higher online offending identified in the United States. It is notable that the United States has not passed significant online child safety reforms at the federal level since 1998 when the Children’s Online Privacy Protection Act was signed into law, imposing restrictions on the online collection of personal data about children under the age of 13. Since then, robust free speech protections as well as industry pressure has stymied efforts to address exponentially increasing rates of technology-facilitated child sexual exploitation and abuse (Citron, 2023). A key distinction between U.S. offenders compared to Australian and U.K. offenders in this study was their heightened engagement with the commercial sexual exploitation of children online. This finding suggests that a laissez-faire approach to online content moderation and user safety not only increases the prevalence of technology-facilitated child sexual exploitation and abuse but also facilitated the development of black markets in which children are sexually abused for financial as well as sexual purposes. Internet regulation is therefore a critically important intervention in order to disrupt and prevent technology-facilitated child sexual exploitation and abuse.

This article also identified the relatively positive psychosocial profile of many online offenders. The findings paint a picture of an offender who appears outwardly respectable and trusted, with access to children, engaged in a range of sexual and predatory online behaviours, while using encryption and other privacy technologies to cover his tracks. These findings cast light on the “offline” behaviour of online offenders, commensurate with the broader “grooming” literature, which finds that offenders may engage in a range of behaviours that deter suspicion and facilitate their access to children (online and offline; Winters & Jeglic, 2017). This information may be useful to mandatory reporters, child protection and law enforcement in early identification and investigation of such men, and assessment of their risk to children.

Our study also found indicators of heightened sexual interest and preoccupation amongst technology-facilitated child sex offenders, including their greater level of engagement with adult pornography and other explicit material. There are currently some initiatives that aim to detect men who are seeking CSAM on adult pornography sites, and refer them to appropriate abuse prevention services (Scanlan et al., 2024). However, our findings suggest that child sexual abuse prevention messaging to pornography consumers could be expanded to include people who are worried about their high level of consumption, or their viewing of deviant content such as bestiality. This cohort of pornography users may also pose a risk to children and may benefit from referral to secondary prevention services.

The online sexual behaviour of offenders in this study (such as paying for online sexual content or subscribing to dating sites) are likely to be visible to online banking and payment services; therefore, these findings may contribute to more effective detection and anti-abuse strategies in the financial sector. Depending on their jurisdiction, some financial entities such as banks have legislative obligations to proactively detect suspicious transactions that are associated with child sexual exploitation and refer these transactions for further investigation. This article suggests that there are patterns of financial behaviour that, while not directly involved in the facilitation of offences against children, may also be indicative of offending behaviour. Nonetheless, men who sexually abuse children who otherwise appear to be prosocial and of good character are very difficult to detect, and there is a need for ongoing policy and practice development to sensitise mandatory reporters, schools, social and welfare services, law enforcement, and criminal justice agencies to the risk posed by this cohort of offenders.

## Supplemental Material

sj-docx-1-jiv-10.1177_08862605251403620 – Supplemental material for Identifying the Demographic and Internet Use Characteristics of Technology-Facilitated Child Sex Offenders Operating in the Australian, U.S. and U.K. General Population Supplemental material, sj-docx-1-jiv-10.1177_08862605251403620 for Identifying the Demographic and Internet Use Characteristics of Technology-Facilitated Child Sex Offenders Operating in the Australian, U.S. and U.K. General Population by Michael Salter, Tyson Whitten, Delanie Woodlock, James Stevenson, Syimah Mat Rani and Deborah Fry in Journal of Interpersonal Violence

## Notes

1. In 2024, significant legislative proposals were advanced at federal level in the United States with the aim of enhancing online child safety and preventing technology-facilitated child sexual exploitation; however, at the time of writing, they have not passed into law. The appetite of the new U.S. administration for internet regulation is unclear.
