# Supplementary material for: Identifying the Demographic and Internet Use Characteristics of Technology-Facilitated Child Sex Offenders Operating in the Australian, U.S. and U.K. General Population
Source: J Interpers Violence. 2026 Mar 3;41(7-8):1544–69. doi: 10.1177/08862605251403620 (PMC12960737; doi:10.1177/08862605251403620)
Supplement: sj-docx-1-jiv-10.1177_08862605251403620 – Supplemental material for Identifying the Demographic and Internet Use Characteristics of Technology-Facilitated Child Sex Offenders Operating in the Australian, U.S. and U.K. General Population [file sj-docx-1-jiv-10.1177_08862605251403620.docx]

| Supplementary Table S1. Weighted unadjusted (U) and adjusted (A) odds of tech-facilitated child sexual exploitation and demographic characteristics. | | | | |
| --- | --- | --- | --- | --- |
|  | | Australia  OR (99% CI) | U.K  OR (99% CI) | U.S  OR (99% CI) |
| Heterosexual | U | 1.74 (0.49 – 6.17) | 0.77 (0.29 – 2.10) | 1.67 (0.54 – 5.11) |
|  | A | 1.72 (0.50 – 5.91) | 0.80 (0.29 – 2.24) | 1.88 (0.56 – 6.32) |
| Had sex with men | U | 1.10 (0.56 – 2.14) | 3.37 (1.76 – 6.44) | 2.67 (1.58 – 4.52) |
|  | A | 1.00 (0.50 – 2.01)^b^ | 2.88 (1.48 – 5.62)^a^ | 1.92 (1.09 – 3.40) |
| Married or living with partner | U | 1.27 (0.72 – 2.22) | 1.02 (0.54 – 1.93) | 1.95 (1.12 – 3.41) |
|  | A | 1.26 (0.69 – 2.30) | 1.23 (0.59 – 2.55) | 1.90 (1.03 – 3.53) |
| Employed | U | 0.94 (0.52 – 1.70) | 4.00 (1.76 – 9.11) | 2.55 (1.35 – 4.83) |
|  | A | 0.96 (0.42 – 2.21) | 3.00 (1.22 – 7.38) | 1.13 (0.52 – 2.42) |
| Bachelor’s degree or higher | U | 0.90 (0.54 – 1.50) | 1.70 (0.92 – 3.12) | 2.97 (1.81 – 4.87) |
|  | A | 0.75 (0.45 – 1.27)^c^ | 1.52 (0.80 – 2.89) | 2.49 (1.37 – 4.51)^a^ |
| Child in household | U | 1.25 (0.74 – 2.11) | 4.21 (2.27 – 7.79) | 3.84 (2.30 – 6.40) |
|  | A | 1.41 (0.78 – 2.54)^b^ | 3.45 (1.76 – 6.75)^a^ | 2.43 (1.39 – 4.23) |
| Works with children | U | 1.95 (1.15 – 3.29) | 4.85 (2.55 – 9.22) | 4.59 (2.73 – 7.72) |
|  | A | 2.03 (1.20 – 3.45) | 3.71 (1.88 – 7.31) | 2.67 (1.54 – 4.64) |
| Residential location | |  |  |  |
| City | U | 1.65 (0.76 – 3.58) | 4.73 (2.02 – 11.12) | 6.80 (3.13 – 14.79) |
|  | A | 1.87 (0.81 – 4.28) | 3.25 (1.38 – 7.68) | 4.76 (2.13 – 10.61) |
| Suburbs | U | 0.71 (0.31 – 1.60) | 2.12 (0.86 – 5.26) | 2.04 (0.85 – 4.88) |
|  | A | 0.77 (0.34 – 1.76) | 2.02 (0.83 – 4.93) | 1.75 (0.71 – 4.30) |
| Regional or rural | U | 1.00 (reference) | 1.00 (reference) | 1.00 (reference) |
|  | A |  |  |  |
| Age | |  |  |  |
| 18 – 34 years | U | 1.46 (0.78 – 2.73) | 2.22 (1.15 – 4.27) | 1.62 (0.96 – 2.73) |
|  | A | 1.29 (0.68 – 2.46) | 1.69 (0.84 – 3.39) | 1.84 (1.06 – 3.20) |
| 35 – 64 years | U | 1.00 (reference) | 1.00 (reference) | 1.00 (reference) |
|  | A |  |  |  |
| 65 years and older | U | 1.79 (0.95 – 3.37) | 0.47 (0.18 – 1.20) | 0.43 (0.19 – 1.01) |
|  | A | 1.84 (0.96 – 3.54) | 0.59 (0.23 – 1.49) | 0.57 (0.24 – 1.39) |
| Household income | |  |  |  |
| Less than US$25,000 | U | 1.16 (0.59 – 2.26) | 0.83 (0.36 – 1.87) | 0.85 (0.35 – 2.08) |
|  | A | 1.07 (0.53 – 2.16) | 0.95 (0.40 – 2.25) | 0.78 (0.31 – 1.96) |
| US$25,000 – US$99,999 | U | 1.00 (reference) | 1.00 (reference) | 1.00 (reference) |
|  | A |  |  |  |
| US$100,000 or more | U | 1.33 (0.76 – 2.35) | 1.16 (0.61 – 2.22) | 2.12 (1.28 – 3.52) |
|  | A | 1.25 (0.69 – 2.24) | 1.03 (0.52 – 2.03) | 1.29 (0.70 – 2.38) |
| U = Unadjusted.  A = Adjusted for age, educational attainment, household income, and residential location.  ^a^ = significantly different from Australian sample at *p* <.05.  ^b^ = significantly different from U.K sample at *p* <.05.  ^c^ = significantly different from U.S sample at *p* <.05. | | | | |

| Supplementary Table S2. Weighted unadjusted (U) and adjusted (A) odds of tech-facilitated child sexual exploitation and online pornography habits. | | | | |
| --- | --- | --- | --- | --- |
|  | | Australia  OR (99% CI) | U.K  OR (99% CI) | U.S.A  OR (99% CI) |
| Watches violent or rough porn | U | 3.77 (2.12 – 6.69) | 7.00 (3.43 – 14.31) | 7.92 (4.52 – 13.87) |
|  | A | 3.90 (2.19 – 6.93) | 6.04 (2.89 – 12.63) | 6.50 (3.65 – 11.57) |
| Watches bestiality | U | 7.34 (3.40 – 15.87) | 12.72 (5.46 – 29.64) | 12.11 (6.43 – 22.80) |
|  | A | 6.73 (3.06 – 14.80) | 17.05 (6.74 – 43.12) | 9.80 (5.02 – 19.13) |
| Purchase sexual content | U | 8.20 (4.45 – 15.11) | 8.36 (4.33 – 16.12) | 13.34 (7.71 – 23.08) |
|  | A | 8.29 (4.49 – 15.32) | 5.98 (2.89 – 12.38) | 8.67 (4.73 – 15.90) |
| Approached by adult selling sexual content online | U | 1.65 (0.97 – 2.81) | 3.38 (1.77 – 6.46) | 5.78 (3.48 – 9.59) |
|  | A | 1.65 (0.97 – 2.79)^c^ | 2.67 (1.39 – 5.14) | 4.83 (2.83 – 8.26)^a^ |
| Approached by child selling sexual content online | U | 6.95 (3.61 – 13.37) | 17.75 (8.25 – 38.18) | 17.30 (9.65 – 30.99) |
|  | A | 7.08 (3.72 – 13.49) | 14.75 (6.86 – 31.70) | 12.17 (6.49 – 22.83) |
| U = Unadjusted.  A = Adjusted for age, educational attainment, household income, and residential location.  ^a^ = significantly different from Australian sample at *p* <.05.  ^b^ = significantly different from U.K sample at *p* <.05.  ^c^ = significantly different from U.S sample at *p* <.05. | | | | |

| Supplementary Table S3. Weighted unadjusted (U) and adjusted (A) odds of tech-facilitated child sexual exploitation and frequency of online activities. | | | | |
| --- | --- | --- | --- | --- |
|  | | Australia  OR (99% CI) | U.K  OR (99% CI) | U.S.A  OR (99% CI) |
| Browse online | U | 0.94 (0.70 – 1.26) | 0.66 (0.48 – 0.91) | 1.01 (0.76 – 1.33) |
|  | A | 1.00 (0.74 – 1.36) | 0.68 (0.48 – 0.98) | 0.92 (0.67 – 1.27) |
| Send emails | U | 0.84 (0.64 – 1.10) | 0.76 (0.58 – 0.99) | 1.15 (0.93 – 1.42) |
|  | A | 0.82 (0.62 – 1.10) | 0.76 (0.57 – 1.02) | 1.02 (0.79 – 1.32) |
| Social media | U | 1.05 (0.85 – 1.31) | 0.89 (0.75 – 1.05) | 1.20 (1.01 – 1.43) |
|  | A | 1.07 (0.85 – 1.34)^b^ | 0.79 (0.65 – 0.96)^a,c^ | 1.04 (0.85 – 1.28)^b^ |
| Online blogs | U | 1.29 (1.08 – 1.55) | 1.29 (1.06 – 1.56) | 1.60 (1.34 – 1.91) |
|  | A | 1.36 (1.12 – 1.66) | 1.09 (0.87 – 1.35) | 1.36 (1.12 – 1.65) |
| Online shopping | U | 1.55 (1.25 – 1.92) | 1.33 (1.03 – 1.70) | 1.81 (1.43 – 2.28) |
|  | A | 1.58 (1.27 – 1.95) | 1.22 (0.87 – 1.44) | 1.48 (1.17 – 1.86) |
| Online banking | U | 1.29 (0.94 – 1.76) | 0.85 (0.67 – 1.07) | 1.35 (1.09 – 1.66) |
|  | A | 1.36 (0.98 – 1.89)^b^ | 0.78 (0.60 – 0.99)^a,c^ | 1.11 (0.89 – 1.39)^b^ |
| Online messaging | U | 1.27 (1.04 – 1.56) | 0.96 (0.81 – 1.14) | 1.40 (1.17 – 1.69) |
|  | A | 1.35 (1.07 – 1.70)^b^ | 0.80 (0.66 – 0.98)^a,c^ | 1.20 (0.97 – 1.48)^b^ |
| Private video chatting | U | 1.28 (1.07 – 1.55) | 1.32 (1.08 – 1.62) | 1.61 (1.35 – 1.93) |
|  | A | 1.37 (1.11 – 1.69) | 1.09 (0.88 – 1.35) | 1.34 (1.09 – 1.64) |
| Livestream self | U | 1.35 (1.17 – 1.56) | 1.49 (1.26 – 1.77) | 1.61 (1.40 – 1.87) |
|  | A | 1.37 (1.17 – 1.61) | 1.30 (1.09 – 1.56) | 1.40 (1.18 – 1.65) |
| Streaming videos | U | 1.05 (0.88 – 1.25) | 1.07 (0.90 – 1.28) | 1.30 (1.09 – 1.56) |
|  | A | 1.09 (0.90 – 1.32) | 0.88 (0.72 – 1.07) | 1.11 (0.89 – 1.39) |
| Romance/dating websites | U | 1.48 (1.25 – 1.75) | 1.56 (1.32 – 1.83) | 1.72 (1.48 – 1.99) |
|  | A | 1.51 (1.24 – 1.84) | 1.37 (1.14 – 1.65) | 1.53 (1.30 – 1.79) |
| Online gaming | U | 1.26 (1.07 – 1.48) | 1.25 (1.06 – 1.47) | 1.48 (1.30 – 1.70) |
|  | A | 1.29 (1.07 – 1.56) | 1.06 (0.86 – 1.32) | 1.30 (1.12 – 1.50) |
| Online porn | U | 1.38 (1.13 – 1.67) | 1.43 (1.17 – 1.74) | 1.54 (1.32 – 1.81) |
|  | A | 1.41 (1.15 – 1.73) | 1.27 (1.04 – 1.55) | 1.42 (1.19 – 1.70) |
| U = Unadjusted.  A = Adjusted for age, educational attainment, household income, and residential location.  ^a^ = significantly different from Australian sample at *p* <.05.  ^b^ = significantly different from U.K sample at *p* <.05.  ^c^ = significantly different from U.S sample at *p* <.05. | | | | |

| Supplementary Table S4. Weighted unadjusted (U) and adjusted (A) odds of tech-facilitated child sexual exploitation and use of social media platforms. | | | | |
| --- | --- | --- | --- | --- |
|  | | Australia  OR (99% CI) | U.K  OR (99% CI) | U.S.A  OR (99% CI) |
| YouTube | U | 1.76 (0.82 – 3.79) | 1.05 (0.54 – 2.03) | 2.34 (1.26 – 4.33) |
|  | A | 2.11 (0.97 – 4.60) | 0.55 (0.27 – 1.11) | 1.65 (0.83 – 3.27) |
| Instagram | U | 2.87 (1.65 – 5.02) | 1.64 (0.89 – 3.01) | 2.66 (1.59 – 4.45) |
|  | A | 3.38 (1.79 – 6.39)^b^ | 0.94 (0.49 – 1.78)^a^ | 1.48 (0.83 – 2.63) |
| Facebook | U | 1.37 (0.73 – 2.55) | 0.96 (0.51 – 1.80) | 1.28 (0.72 – 2.28) |
|  | A | 1.42 (0.75 – 2.68) | 1.06 (0.55 – 2.05) | 1.08 (0.59 – 1.98) |
| Snapchat | U | 2.06 (1.24 – 3.44) | 2.09 (1.09 – 4.00) | 3.50 (2.14 – 5.73) |
|  | A | 2.19 (1.20 – 4.00) | 1.15 (0.54 – 2.46) | 2.37 (1.40 – 4.04) |
| Facebook messenger | U | 1.36 (0.81 – 2.30) | 1.10 (0.59 – 2.03) | 1.65 (1.01 – 2.70) |
|  | A | 1.46 (0.85 – 2.51) | 1.00 (0.54 – 1.86) | 1.40 (0.83 – 2.36) |
| Tik Tok | U | 1.53 (0.90 – 2.58) | 2.68 (1.44 – 4.98) | 2.49 (1.53 – 4.06) |
|  | A | 1.76 (0.94 – 3.29) | 1.69 (0.86 – 3.33) | 1.60 (0.93 – 2.74) |
| WhatsApp | U | 1.60 (0.96 – 2.68) | 1.80 (0.93 – 3.49) | 4.75 (2.88 – 7.84) |
|  | A | 1.67 (0.96 – 2.90) | 1.42 (0.73 – 2.78) | 2.92 (1.70 – 5.01) |
| Twitter | U | 1.26 (0.75 – 2.11) | 1.18 (0.63 – 2.21) | 2.81 (1.72 – 4.59) |
|  | A | 1.26 (0.74 – 2.12) | 0.92 (0.48 – 1.76) | 1.70 (1.02 – 2.85) |
| Discord | U | 1.29 (0.68 – 2.43) | 2.87 (1.35 – 6.12) | 2.36 (1.26 – 4.42) |
|  | A | 1.27 (0.66 – 2.45) | 1.79 (0.80 – 3.99) | 1.62 (0.84 – 3.13) |
| Skype | U | 2.96 (1.68 – 5.19) | 1.39 (0.57 – 3.43) | 3.90 (2.24 – 6.81) |
|  | A | 2.63 (1.52 – 4.57) | 1.03 (0.43 – 2.50) | 2.67 (1.48 – 4.81) |
| Viber | U | 1.75 (0.64 – 4.80) | 1.79 (0.54 – 6.00) | 4.57 (1.72 – 12.16) |
|  | A | 2.07 (0.71 – 6.08) | 1.52 (0.79 – 2.89) | 2.37 (1.29 – 4.32) |
| U = Unadjusted.  A = Adjusted for age, educational attainment, household income, and residential location.  ^a^ = significantly different from Australian sample at *p* <.05.  ^b^ = significantly different from U.K sample at *p* <.05.  ^c^ = significantly different from U.S sample at *p* <.05. | | | | |

| Supplementary Table S5. Weighted unadjusted (U) and adjusted (A) odds of tech-facilitated child sexual exploitation and use of privacy software and anonymity tools. | | | | |
| --- | --- | --- | --- | --- |
|  | | Australia  OR (99% CI) | U.K  OR (99% CI) | U.S.A  OR (99% CI) |
| Any privacy software | U | 2.24 (1.27 – 3.94) | 3.07 (1.50 – 6.29) | 6.36 (3.35 – 12.07) |
|  | A | 2.18 (1.15 – 4.12) | 2.05 (0.97 – 4.35) | 3.97 (2.05 – 7.69) |
| TOR | U | 2.55 (1.00 – 6.52) | 1.99 (0.70 – 5.67) | 5.86 (3.16 – 10.85) |
|  | A | 2.43 (0.92 – 6.44) | 1.32 (0.44 – 3.99) | 3.27 (1.75 – 6.10) |
| VPN | U | 1.99 (1.18 – 3.37) | 1.44 (0.73 – 2.83) | 1.97 (1.20 – 3.24) |
|  | A | 1.92 (1.12 – 3.27) | 1.09 (0.54 – 2.19) | 1.44 (0.85 – 2.43) |
| Telegram | U | 6.13 (3.55 – 10.60) | 4.56 (2.30 – 9.06) | 6.71 (3.97 – 11.33) |
|  | A | 5.40 (3.06 – 9.56) | 3.03 (1.44 – 6.35) | 3.85 (2.19 – 6.77) |
| Signal | U | 2.19 (1.21 – 3.99) | 5.69 (2.46 – 13.19) | 8.02 (4.20 – 15.29) |
|  | A | 1.67 (0.89 – 3.13)^c^ | 3.61 (1.49 – 8.75) | 4.21 (2.19 – 8.10)^a^ |
| WhatsApp | U | 2.42 (1.44 – 4.07) | 1.66 (0.90 – 3.05) | 5.40 (3.26 – 8.94) |
|  | A | 2.42 (1.33 – 4.40) | 1.10 (0.57 – 2.13)^c^ | 3.37 (1.96 – 5.81)^b^ |
| Element | U | 5.40 (2.27 – 12.85) | 4.58 (1.19 – 17.61) | 8.69 (3.91 – 19.32) |
|  | A | 3.57 (1.36 – 9.32) | 2.80 (0.62 – 12.61) | 3.96 (1.64 – 9.55) |
| Hive | U | 2.21 (0.87 – 5.62) | 4.32 (1.22 – 15.34) | 8.14 (3.54 – 18.73) |
|  | A | 1.49 (0.49 – 4.52) | 1.46 (0.77 – 2.79) | 2.36 (1.28 – 4.36) |
| Private relay (Safari) | U | 2.10 (0.67 – 6.59) | 0.45 (0.06 – 3.22) | 3.35 (1.46 – 7.69) |
|  | A | 1.90 (0.60 – 5.99) | 0.31 (0.04 – 2.38) | 1.68 (0.74 – 3.78) |
| Owns cryptocurrency | U | 1.81 (1.09 – 3.03) | 2.64 (1.39 – 5.04) | 4.44 (2.65 – 7.46) |
|  | A | 1.86 (1.07 – 3.23) | 1.77 (0.91 – 3.45) | 2.72 (1.51 – 4.87) |
| Uses cryptocurrency | U | 3.06 (1.70 – 5.52) | 4.69 (2.26 – 9.75) | 7.23 (4.31 – 12.13) |
|  | A | 2.71 (1.49 – 4.94) | 2.86 (1.28 – 6.40) | 4.28 (2.36 – 7.75) |
| U = Unadjusted.  A = Adjusted for age, educational attainment, household income, and residential location.  ^a^ = significantly different from Australian sample at *p* <.05.  ^b^ = significantly different from U.K sample at *p* <.05.  ^c^ = significantly different from U.S sample at *p* <.05. | | | | |
